# Nutritional Composition, Pharmacological Properties, and Industrial Applications of *Myrciaria dubia*: An Undiscovered Superfruit

**DOI:** 10.1002/fsn3.70331

**Published:** 2025-05-23

**Authors:** Sammra Maqsood, Muhammad Tayyab Arshad, Ali Ikram, Kodjo Théodore Gnedeka

**Affiliations:** ^1^ National Institute of Food Science and Technology University of Agriculture Faisalabad Pakistan; ^2^ University Institute of Food Science and Technology The University of Lahore Lahore Pakistan; ^3^ Togo Laboratory: Applied Agricultural Economics Research Team (ERE2A) University of Lomé Lome Togo

**Keywords:** antioxidants, Camu Camu, carotenoids, functional food

## Abstract

The diminutive recognized fruit *Myrciaria dubia*, innate to the Amazon, has only newly attained nutritional superstar status due to its dense content of bioactive compounds. Despite its potential, processing concerns, unsustainable fabrication, and a deficiency of experimental trials hinder its broader application. This review aimed to highlight the nutritional composition, pharmacological properties, and industrial applications of *Myrciaria dubia*, emphasizing its potential as a functional ingredient in various sectors. The bioactive profile of *Myrciaria dubia* comprises vitamin C, polyphenols, flavonoids, anthocyanins, and ellagic acid, contributing to its metabolic, anti‐inflammatory, antioxidant, and antibacterial actions. Comparative analyses suggest its bioavailability and beneficial activity surpass those of other fruits, making it a strong candidate for functional foods, beverages, cosmetics, and pharmaceuticals. Given its promising attributes, further investigation and biotechnological advancements are necessary to enhance its bioactive content and ensure its sustainable integration into global markets. This review brings to the forefront the importance of *Myrciaria dubia* as a tool for addressing modern health challenges while supporting biodiversity conservation.

## Graphical abstract

This review highlights the remarkable potential of *Myrciaria dubia*, an underutilized Amazonian fruit, as a source of bioactive compounds with diverse applications in functional foods, beverages, cosmetics, and pharmaceuticals. Our review not only underscores the fruit's potential in addressing global health concerns but also emphasizes the need for biotechnological innovations to enhance its bioactive content and facilitate its integration into international markets.

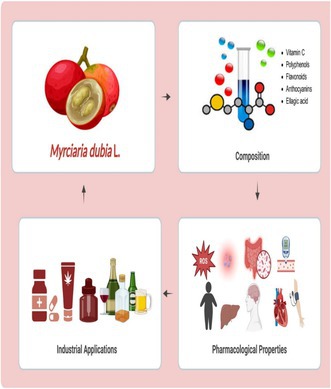

## 1 Introduction

Bioactive composites in the shape of chemical molecules present in food have turned into strong tools for ornamental wellness and minimizing the risk of long‐term ailments (González 2020). Glucosinolates, polyphenols, carotenoids, and alkaloids are some of the many chemical complexes that can prevent or reduce conditions like diabetes, cancer, cardiovascular disease, and neurological disorders (Banwo et al. 2021; González 2020). Since inflammation and oxidative stress play an essential role in leading to chronic diseases, the said materials are considered to possess healing properties because they are anti‐inflammatory, antibacterial, and antioxidant in nature (Kris‐Etherton et al. 2002). An example is the flavonoid and polyphenol, which improves endothelial function and decreases atherosclerosis occurrence (Walia et al. 2019). As part of health development and disease anticipation, individuals are progressively including bioactive composites into their diets, mainly by consuming extra fruits, vegetables, and whole grains (Martirosyan and Miller 2018).

Experts have exposed that bioactive phytochemicals in whole foods use a more noteworthy effect associated with isolated supplements, thus one should eat a diverse diet rich in plant foods (van Breda and de Kok 2018). Bioactive phytochemicals, like glucosinolates in Brassicaceae vegetables and selenium‐rich crops, demonstrate that these minerals play a double role in nutrition and disease anticipation (Raiola et al. 2017; Newman et al. 2019). Additional research on the activity of bioactive chemicals is illuminating their possible use in functional nutrition as an association among food science and medicine, and these molecules have a significant position in the scientific area. Mysterious fruits have emerged due to the worldwide inventiveness of healthier dietaries (González‐Aguilar et al. 2008). The bioactive materials in the fruit are abundant; these naturally have their place in an area, and most of them get unexploited. The phytochemicals found in profusion in these fruits are vitamins, carotenoids, polyphenols, and flavonoids. Their robust antibacterial, anti‐inflammatory, and antioxidant activities are advantageous for disease anticipation and health elevation (Gupta et al. 2013; Murthy and Bapat 2020). Though owning interesting nutritional characteristics, numerous of these fruits are yet to be widely studied because of poor commercial fabrication, ignorance, and addition in mainstream food systems (Meena et al. 2022).

Health benefits have been displayed by bioactive composites found in wild fruits such as wild annonas, bael, and *Myrciaria dubia*. Castro et al. (2018) and Sousa et al. (2021), for occurrence, stated that the greater levels of vitamin C, anthocyanins, and ellagic acid found in *Myrciaria dubia* improve its antioxidant and anti‐inflammatory activities. As per Sharma et al. (2022), bael belongs to the class of nutraceutical drugs as it comprises coumarins and flavonoids, which are antiviral and hepatoprotective in their accomplishment. What drives this trend of high interest in the said fruits' prospect of functional food ingredients to feed the demand for natural product‐based products with the attributes of promoting health, Donno and Turrini (2020). Research in underutilized fruit preservation, utilization, and marketing has been increased Arshad et al. (2025). These comprise encouraging sustainable harvesting methods, conducting value‐added goods investigations, and enhancing postharvest technologies (Meghwal et al. 2022; Dasila and Singh 2022). Moreover, investigations on fruits such as *Myrciaria dubia* and *Elaegnus latifolia* have established their prospective to avert cardiovascular diseases and long‐lasting diseases such as diabetes because of their bioactive content (Hegazy et al. 2019; Neves et al. 2017). In addition to updating individuals in poor zones about the necessity for food security and economic feasibility, these fruits can also lead to an extra differentiated diet. Native to the Amazon rainforest, the petite cherry‐sized fruit *Myrciaria dubia* (*Myrciaria dubia*) has captivated the masses because of its incredible bioactive and nutritional properties (Meena et al. 2022). Another of the ironic natural sources of vitamin C, *Myrciaria dubia* comprises an even broader spectrum of phytochemicals that make it all the more auspicious as a functional food and nutraceutical. Further being advantageous to the health of human beings, the bioactive composites contained in this fruit render it an ecofriendly produce (Peduruhewa et al. 2021).

*Myrciaria dubia* cultivates on riverbanks and floodplains in Colombia, Brazil, and Peru in the Amazon basin. The medicinal solicitations of the fruit have been centuries old, prior to it becoming worldwide renowned (Donno et al. 2018). The antibacterial, anti‐inflammatory, and antioxidant properties of this food are mainly attributed to its high content of bioactive compounds, including organic acids, phenolics, and flavonoids (Neves et al. 2017). There are limited fruits that are as great in vitamin C as *Myrciaria dubia*. A rough estimation of 2000 to 3000 mg per 100 g of fresh pulp has been quoted by Langley et al. (2015). Their antioxidant activity is improved by the occurrence of ellagic acid, anthocyanins, and carotenoids, alongside a great level of ascorbic acid. Pereira‐Netto (2018) adds that other fruits have also been used to treat disorders linked to oxidative stress, comprising diabetes, cardiovascular diseases, and neurodegenerative disorders. Within a short period, *Myrciaria dubia* has transitioned from being a communal meal in just one area to an international phenomenon. Singh et al. (2018) detected that it is a valuable representative in the field of health foodstuffs due to its bioactive nature, as it is valuable in dietary supplements, cosmetics, and functional drinks. Natural antioxidant sources and sustainable agri‐products are inordinate demand worldwide; therefore, their worth has been raised (Castro et al. 2018).

*Myrciaria dubia* assists more than your well‐being; it can assist you in meeting your sustainability and biodiversity purposes (Langley et al. 2015). Its fabrication supports international efforts to save Amazon's subtle ecosystem. It also assists local communities economically (Yunis‐Aguinaga et al. 2016). As Conceição et al. (2019) described, developments in agricultural performance and processing technology can hypothetically improve its value chain while at the same time decreasing its environmental footprint. In conclusion, *Myrciaria dubia* assimilates traditional knowledge and contemporary science to propose a bioactive element source that is robust and versatile. An increasing number of persons are beginning to value it for the means in which it may assist with prevailing health problems and foster social and environmental pliability. As experts work to classify its bioactive potential, subsequent investigations will shed light on its fitness and wellness usage. In the modern world, the increasing occurrence of chronic diseases has intensified the demand for health‐endorsing foods, chiefly plant‐based choices like leafy green vegetables. These nutrient‐rich foodstuffs, like *Cnidoscolus aconitifolius* (Chaya or tree spinach), are packed with phytochemicals and vital nutrients, proposing important potential to decrease the risk of cardiovascular and neurodegenerative illnesses while refining overall well‐being and quality of life (Panghal et al. 2021). In recent years, the increasing demand for healthy food selections has driven the progress of functional foods, which comprise benefits beyond straightforward nutrition, such as disease prevention and general well‐being. Functional foodstuffs comprising whole, fortified, or enhanced products are attaining admiration because of their bioactive constituents like antioxidants, polyphenols, and probiotics (Tufail et al. 2025a; Chhikara and Panghal 2022). This shift reflects a wider societal attention on combating lifestyle‐associated illnesses through dietary interventions. This review discovers *Myrciaria dubia* (Camu Camu), an underutilized superfruit, emphasizing its nutritional composition, pharmacological properties, and potential industrial applications as a functional food (Anil et al. 2022).

In this review, we will inspect the bioactive biochemical conformation of *Myrciaria dubia*, a fruit with prospective pharmaceutical solicitations, nutritional determinations, and medicinal solicitations. This review aimed to highlight a fruit superior to most concerning bioactive contents, human health valuation, comparison to other fruits, chances, and challenges of sustainable farming, processing, and usage in the cosmetics, nutraceuticals, and functional food industries by amalgamating existing investigation.

## 2 Nutritional and Bioactive Profile of *Myrciaria dubia*

**FIGURE 1:**
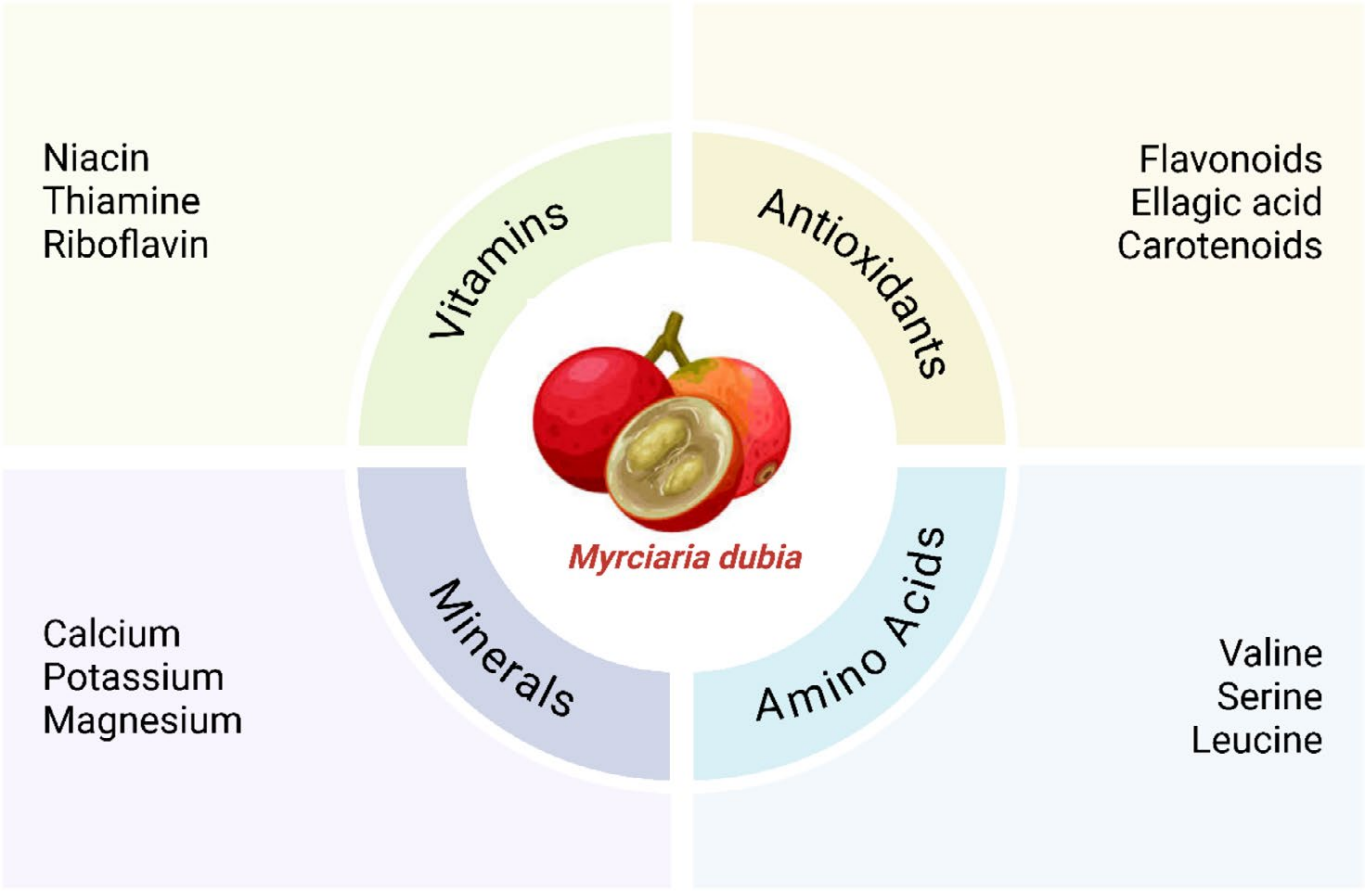
Nutrition composition of *Myrciaria dubia*.

*Myrciaria dubia*, which is an innate fruit of the Amazon, is well known for comprising bioactive composites, particularly with great amounts of vitamin C, polyphenols, and antioxidants (Figure 1) (Conceição et al. 2019). These are mainly due to these bioactive composites which exhibit antioxidant, anti‐inflammatory, and antibacterial properties in nature. A precipitate of the chief bioactive compounds found in *Myrciaria dubia* is presented, as well as facts about its nutritional value and probable health assistances. *Myrciaria dubia* varies from other citrus fruits because of its extraordinary vitamin C level. It has 2800 to 3000 mg of vitamin C for each 100 g. This can definitely surpass the recommended daily minimum of 75 mg for ladies and 90 mg for males (Akter et al. 2011). Collagen amalgamation, immune function, and oxidative stress decrease all rely on this powerful antioxidant while decreasing oxidative stress and inflammation (Tufail et al. 2025b). In addition to being a supporter of skin integrity and immunological health, this also improves the healing of wounds. *Myrciaria dubia* is rich in numerous phenolic composites such as flavonoids, ellagic acid derivatives, and proanthocyanidins. These help to improve its high antioxidant activity through free radical scavenging and prevention of oxidative impairment.

The anticancer and anti‐inflammatory properties of ellagic acid have caused ellagic acid to become well recognized (Fracassetti et al. 2013). Grigio, Moura, Carvalho, et al. (2021) and Grigio, Moura, Chagas, et al. (2021) exposed that proanthocyanidins have positive effects on the cardiovascular system, such as decreasing blood pressure and improving vascular performance. Cardiovascular defense, anti‐inflammatory, anticancer, and antioxidant are certain of the health benefits. *Myrciaria dubia* comprises various flavonoids, and these include anthocyanins, kaempferol, and quercetin. The anti‐inflammatory, anticancer, and antioxidant properties of all these are well recognized. Flavonoids have been described by Costa et al. (2013) to aid in cardiovascular diseases by improving blood flow and decreasing oxidative damage to the blood vessels. Their anticancer properties, reduced risk of cardiovascular problems, and improved brain function are their main benefits.

*Myrciaria dubia* is tannin amusing, a group of polyphenolic substances that are known to have astringent properties. Tannins have been exposed by investigations to constrain the growth of microbes, thus both establishing antioxidants and antibacterials (Costa et al. 2013). Health benefits include traits that combat microbes, inflammation, and free radicals. The carotenoids, beta‐carotene and lutein present in *Myrciaria dubia* improve the antioxidant capability of the fruit but to a lesser degree compared with other instances. These are the forerunners to vitamin A and assist with immune function, skin defense, and eye complications (Bataglion et al. 2015). The fruit protects against macular degeneration, has healthier skin, and increases immunity. Amino acids in *Myrciaria dubia* include arginine, glycine, and glutamine, further enhancing the nutrient potential. Such amino acids support functions of cellular repair, responses by the immune system, and synthesis of proteins. The health benefits are the fruit promotes immunological function, helps repair damaged tissue, and supports muscle health (Table 1) (Røsjø et al. 2020).

**TABLE 1:** Composition of *Myrciaria dubia* fruit (Akter et al. 2011; Rodrigues et al. 2020).

Content	Value (per 100 g)
Energy	52 kcal
Carbohydrates	13.1 g
Dietary fiber	3.3 g
Protein	0.8 g
Fat	0.2 g
Vitamin C	2800–3000 mg
Calcium	16 mg
Iron	1.0 mg
Potassium	195 mg

### 2.1 Polyphenols and Flavonoids

Flavonoids and polyphenols, two of the plant‐based bioactive substances abundant in *Myrciaria dubia*, have been shown to possess critical health‐promoting properties. Most of the fruit's potent anti‐inflammatory and antioxidant qualities are attributed to these chemicals (Castro et al. 2018). Flavonoids, quercetin, catechins, and anthocyanins contribute to the bioactivity of *Myrciaria dubia*; important polyphenols for this plant are tannins, gallic acid, and ellagic acid. These materials have their biological possessions strengthened through their synergy (García‐Chacón et al. 2023; Fidelis, de Oliveira, et al. 2020; Fidelis, do Carmo, et al. 2020). *Myrciaria dubia* has active composites like flavonoids and polyphenols that are operative antioxidants, and these decrease oxidative stress by eliminating free radicals and avoidance the manufacture of reactive oxygen species (ROS). Cancer, cardiovascular disease, dementia, and other important chronic disorders associated with DNA mutation, cellular impairment, and lipid peroxidation comprise cancer (Chang et al. 2025; Grigio et al. 2017; Fujita et al. 2015). Both Do et al. (2021) and Langley et al. (2015) have noted *Myrciaria dubia*'s capacity to stimulate Nrf2 signaling pathways and restore endogenous antioxidants such as glutathione, hereafter strengthening cellular protection against oxidative assaults. By metal ion chelation and superoxide anion efficient scavenging, polyphenols, tannins, and ellagic acid have shown promise in lowering oxidative impairment (Barbalho et al. 2022; Musachio et al. 2024). *Myrciaria dubia*'s flavonoid antioxidant activity, more particularly, quercetin's capability to scavenge reactive oxygen species (ROS) is thought to be a consequence of electron capability (García‐Chacón et al. 2023). By affecting significant inflammatory pathways, the flavonoids and polyphenols present in *Myrciaria dubia* produce strong anti‐inflammatory activities. As Fujita et al. (2017) and Do et al. (2021) disclose, such drugs limit the molecular level manufacture of inflammatory cytokines including cyclooxygenase‐2 (COX‐2), tumor necrosis factor‐alpha (TNF‐α), and interleukin‐6 (IL‐6). These substances not only lessen inflammation but also regulate NF‐κB signaling pathways, which assist most organs in yielding fewer inflammatory reactions, as stated by Fidelis, de Oliveira, et al. (2020) and Fidelis, do Carmo, et al. (2020). *Myrciaria dubia* bioactives have anti‐inflammatory possessions that aggravate autoimmune illnesses and metabolic syndromes associated with persistent inflammation. Ellagic acid overwhelms inflammatory mediator activation; quercetin decreases oxidative stress in inflammatory illnesses; and anthocyanins do too (Donado‐Pestana et al. 2018; Hernández et al. 2011). *Myrciaria dubia* polyphenols and flavonoids interrelate to upsurge the therapeutic promise of the plant (Han et al. 2025). Not only from defensive molecules and cells, their antioxidant and anti‐inflammatory possessions tend to aid in tissue healing while preserving immunological homeostasis (Castro et al. 2020; Akter et al. 2011). A communal side effect of chronic medical conditions is oxidative stress‐induced inflammation; this notion assists in decreasing such inflammation. *Myrciaria dubia*'s bioactive constituents, either ingested or encompassed into functional foods as a component, naturally defend against oxidative stress and inflammation, consequently refining general health (Donado‐Pestana et al. 2021).

### 2.2 Anthocyanins and Ellagic Acid

Common fruits counting *Myrciaria dubia* (Fujita et al. 2013) comprise bioactive composites named anthocyanins and ellagic acid. The anticipation of prolonged illnesses and cellular protection depends much on the anti‐inflammatory and antioxidant properties of these complexes. Frequently found in maximum fruits, anthocyanins are a group of water‐soluble flavonoids that yield red, purple, and blue hues (Fidelis et al. 2018). Well‐explored and with favorable effects on human health, these compounds' anti‐inflammatory and antioxidative properties have been exposed (Castro et al. 2018). One of the main reasons for cell deficiency is oxidative stress, which aids in averting the onset of prolonged diseases comprising diabetes, cardiovascular disease, and neurological illnesses. Anthocyanins improve the antioxidant protection system of the body, which consists of superoxide dismutase and glutathione peroxidase, thus supporting wide‐ranging investigations (Fujita et al. 2015; García‐Chacón et al. 2023). Synergistic interactions with vitamin C in *Myrciaria dubia* improve the antioxidative properties of the anthocyanins, henceforth rising their later capability. Through the antioxidative activity, the defense of proteins and DNA against oxidative damage reduces the probability of long‐lasting inflammation and related disorders such as cancer and metabolic syndrome (Fracassetti et al. 2013; Vargas et al. 2024). Ellagic acid is also a regular polyphenol with the potential to defend cell health. Ellagic acid has antioxidant, anti‐inflammatory, and antimutagenic activity in fruitlets like *Myrciaria dubia*, which is a recognized fact. Subsequently, ellagic acid scavenges free radicals and chelates metal ions, and the biochemical removes oxidative stress (Langley et al. 2015; Musachio et al. 2024). Ellagic acid is proficient in remodeling cellular pathways associated with apoptosis and inflammation with amazement. The initiation of pathways that play a key role in long‐lasting inflammatory diseases, like mitogen‐activated protein kinase (MAPK) and nuclear factor kappa B (NF‐κB), is inhibited. Do et al. (2021) resolved that the Nrf2 pathway also induces the fabrication of detoxifying enzymes, which would improve the defense of the body against oxidative stress. The capability to moderate epigenetic functions, that is, to modify the expression of genes in terms of apoptosis, inflammation, and cancer development, is yet another effect of ellagic acid. A case in point for its anti‐inflammatory nature is that it is capable of hindering the construction of cytokines leading to inflammation, such as TNF‐α and IL‐6 (Barbalho et al. 2022). Because of their antioxidant and anti‐inflammatory qualities, anthocyanins and ellagic acids play a critical role in resisting prolonged diseases. By improving the endothelial process, declining pressure levels, and constraining oxidation LDL, these will reduce cardiovascular diseases (Fujita et al. 2017).

Assmann et al. (2021) have described that these composites exert neuroprotective activities in models of neurodegenerative illnesses through the modulation of neuroinflammation and inhibition of oxidative impairment to neurons. Type 2 diabetes and obesity are two syndromes that can be cured by anthocyanins and ellagic acid's capability to decrease inflammation in adipose tissue as well as improve insulin sensitivity. Control of effect for hyperglycemia and evasion of metabolic syndrome problems is significant, as designated by Nascimento et al. (2013) and Akter et al. (2011). The anti‐inflammatory and antioxidant properties of ellagic acid and anthocyanins are strong cellular defenses, and the two composites (Anthocyanins and Ellagic acid) form the core of fruits like *Myrciaria dubia*. Their effectiveness accentuates their power to modify significant metabolic purposes, which could be translated into the anticipation and control of chronic situations. Novel and promising information on the prospective medicinal and gastronomic application of such bioactives is being developed from current investigation, mainly when targeted at fruits that are native to the Amazon, like *Myrciaria dubia*.

A disproportion among the body's reactive oxygen species (ROS) and its antioxidant resistances describes oxidative stress; numerous chronic diseases, comprising as cancer, neurological disorders, and cardiovascular illnesses, have a reflective influence on this disorder. Antioxidants' function in warding off this disease has grown clearer in light of the mounting investigation showing that these can both avert and treat oxidative stress (Chang et al. 2019). According to Castro et al. (2018), bioactive composites present in fruits and plants have antioxidant properties that could assist in the prevention of oxidative stress‐associated illnesses. To defend cells and tissues from free radical impairment, antioxidants function as a buffer. These molecules produce oxidative stress if they are not controlled. This stress can lead to inflammation, cellular aging, and the deterioration of prolonged diseases. The antioxidant phytochemicals present in numerous plant‐based diets have expanded a lot of consideration recently. According to Sagar et al. (2018), these ingredients incorporate vitamins, carotenoids, flavonoids, and polyphenols. Their capability to chelate metal ions, change numerous enzymatic processes, and scavenge‐free radicals marks them vital for oxidative stress regulation (Chang et al. 2019). Antioxidant activity with therapeutic potential for evading oxidative stress‐associated diseases has been established in a substantial quantity of studies. Take, for instance, the polyphenols present in certain tropical fruits and berries like Nanche ( *Byrsonima crassifolia* ) and Camu Camu (*Myrciaria dubia*), alongside citrus fruits. These materials have shown extraordinary antioxidant properties, decreasing oxidative stress markers in both laboratory and animal investigations (Salehi et al. 2020; García‐Chacón et al. 2023). These physiologically active substances not only neutralize reactive oxygen species (ROS), but they also stimulate the Nrf2 pathway and other endogenous antioxidant protection mechanisms that the body uses to keep cellular redox equilibrium (Martins et al. 2016). Oxidative stress is implicated in the pathogenesis of CVDs, which are one of the major causes of death worldwide. ROS is highly involved in the pathophysiology of atherosclerosis through lipid peroxidation, inflammation, and endothelial dysfunction. By reducing oxidative damage to proteins, lipids, and DNA, antioxidants have been shown to decrease these effects and thereby reduce the risk of cardiovascular events (Nick 2006). Increasing nitric oxide availability and lessening arterial stiffness is crucial for plant‐derived bioactives such as the flavonoids found in fruits such as grapes and apples in improving vascular health (Pereira‐Netto 2018). For instance, Neri‐Numa et al. (2018) found that antioxidants in tropical fruits, including the açai berry and other wild Brazilian fruits, significantly reduce inflammation and oxidative stress, two primary factors causing CVD. These fruits, which contain high levels of phenolic compounds, may be useful in natural remedies to prevent the development of cardiovascular disease by preventing the oxidative damage associated with hypertension and atherosclerosis (Table 2).

**TABLE 2:** Compounds of *Myrciaria dubia* their role and benefits.

Compound	Value	Benefit	Role	Reference
Vitamin C	2800–3000 mg per 100 g	Immune system enhancement, antioxidative properties, skin health, and wound healing	Major role in immunity is to reduce oxidative stress collagen synthesis and support overall immune response	Neves et al. (2015)
Polyphenols	~50–200 mg per 100 g	Antioxidant, anti‐inflammatory, and anticarcinogenic effects	Reduces oxidative damage, protects cells from inflammation, and supports cellular health	Rodrigues et al. (2020)
Flavonoids	1.2–2.0 mg per 100 g	Reduces risk of cardiovascular diseases, antioxidant and anti‐inflammatory properties	It protects vascular health, reduces blood pressure, and supports anti‐inflammatory pathways	Castro et al. (2020)
Anthocyanins	~0.05–0.1 mg per 100 g	Antioxidant, anti‐inflammatory, supports cardiovascular health	It protects against oxidative stress and inflammation and supports eye and heart health	Azevedo et al. (2019)
Ellagic acid	~2.8 mg per 100 g	Antioxidant, anticancer, and antiviral properties	It protects cells from oxidative damage, reduces inflammation, and helps prevent DNA mutations	Grigio, Moura, Carvalho, et al. (2021), Grigio, Moura, Chagas, et al. (2021)
Tannins	~0.2–1.0 mg per 100 g	Antioxidant, antimicrobial, anti‐inflammatory, and anticarcinogenic effects	Reduces oxidative stress, supports immune health, and protects against bacteria and viruses	García‐Chacón et al. (2024)
Carotenoids	~0.1–1.5 mg per 100 g	Supports vision, skin health, and antioxidant activity	Reduces free radical damage and supports eye health by maintaining the retina and lens integrity	Castro et al. (2018)
Fiber	~2.0 g per 100 g	It aids digestion, promotes gut health, and supports weight management	It enhances gut motility, reduces blood cholesterol, and improves microbiota balance	Ikram et al. 2024; da Silva et al. (2023)
Sugar (Fructose)	~3.0–4.0 g per 100 g	It provides quick energy and helps with glucose metabolism	It is an immediate energy source and supports brain and muscle function	Akter et al. (2011)
Protein	~0.6–0.8 g per 100 g	Supports muscle repair, immune function, and overall growth	It contributes to cellular repair and enzyme activity and supports immune defense	do Amaral Souza et al. (2019)
Minerals (Calcium, Iron, Potassium)	Varies (~40 mg Calcium, ~2 mg Iron, ~200 mg Potassium)	Supports bone health, oxygen transport, and electrolyte balance	Essential for bone mineralization, oxygen transport in blood, and nerve/muscle function	Fidelis et al. (2018)
Citric acid	~10–15 g per 100 g	It aids digestion, supports kidney health and has antioxidant properties	It supports digestion, regulates pH, and prevents kidney stone formation	Nascimento et al. (2013)
Resveratrol	~0.5 mg per 100 g	Antioxidant, anti‐inflammatory, and potential anticancer properties	Reduces oxidative stress, protects heart health, and may aid in longevity	García‐Chacón et al. (2023)

### 2.3 Vitamin C

One of the Amazonian fruits most valued for its vitamin C content is *Myrciaria dubia*, scientifically known as *Myrciaria dubia*, a nutritional treasure for health‐conscious consumers (Cunha‐Santos et al. 2022). Compared with oranges or acerola cherries, this fruit has an amazingly greater vitamin C content, up to 2800 mg per 100 g of pulp (Neves et al. 2015). Ascorbic acid is another popular name for vitamin C, and it has numerous effects on physiology, such as tissue repair, oxidative stress reduction, and immune defense systems (Castro et al. 2018). Vitamin C is indispensable in improving immunity as it stimulates the production of lymphocytes, which are crucial white blood cells that produce adaptive immunity, and helps increase the function of phagocytes, which engulf and eliminate infectious organisms (Azevedo et al. 2019). In addition, it facilitates interferon synthesis, proteins that trigger the immunity cells and inhibit viral replication. Vitamin consumption from nutrient‐dense foodstuffs such as *Myrciaria dubia* can result in augmented resistance to infection and improved immunity. Vitamin C‐rich foods such as *Myrciaria dubia* could decrease the severity of cold symptoms, according to investigation. Severity and duration that a cold remains in the body are both reduced by these illnesses (Rodrigues et al. 2020). Reactive oxygen species (ROS) threaten vitamin C, interpreting it a powerful antioxidant that protects cells against oxidative damage. Through cellular metabolism and as a reaction to environmental stresses such as pollution and UV radiation, slow‐moving particles recognized as reactive oxygen species (ROS) are formed (Howe et al. 2006). Attributing to Souza et al. (2017), oxidative stress, which might be activated by a disproportion in the production of ROS in the body, is associated with prolonged diseases like diabetes, cardiovascular diseases, and neurological conditions. *Myrciaria dubia*'s high contented of vitamin C is, to a large extent, to blame for its astonishing antioxidant effectiveness, which decreases these threats (Neves et al. 2015). Vitamin C's antioxidant capability to regenerate makes it an effective defense against free radicals. Grigio, Moura, Carvalho, et al. (2021) and Grigio, Moura, Chagas, et al. (2021) exposed that the intake of *Myrciaria dubia* juice meaningfully drops the body's oxidative stress indicators. In sum, it encourages collagen synthesis, which is critical for wound healing, skin elasticity, and circulatory well‐being (Zhang et al. 2024).

The vitamin C from *Myrciaria dubia* is extremely bioavailable, as the body willingly absorbs and uses it. *Myrciaria dubia* relishes a physiological benefit over synthetic vitamin C supplements owed to its synergistic exploit of natural bioactive composites (Neves et al. 2015). The fruit's ascorbic acid content remains wide‐ranging even after being exposed to certain processing procedures, which marks it as a practicable functional component for treatment in food and drink (Rodrigues et al. 2020). There is a suggestion that eating vitamin C ironic foods on a everyday basis can reduce the likelihood of developing prolonged diseases. Its anti‐inflammatory and antioxidant possessions render it a valuable resource in the fight in contradiction of cancer, Type 2 diabetes, and atherosclerosis (Azevedo et al. 2019). *Myrciaria dubia* can develop a functional food that recover public health acknowledgments to its extraordinary vitamin C contented. *Myrciaria dubia*, being a source of vitamin C, has outstanding recompenses for lowering oxidative stress and ornamental immune system function. It is a wonderful nutritional constituent that keeps you healthy and repels chronic diseases; it has outstanding bioavailability and roles in synergy with other bioactives (Neves et al. 2015). We will continue increasing our knowledge about its medicinal potential and how to integrate it into pharmaceutical and nutraceutical preparations.

## 3 Health Benefits of *Myrciaria dubia*

### 3.1 Antioxidant Effect

Also, neurodegenerative diseases such as Alzheimer's, Parkinson's, and Huntington's have been associated with oxidative stress. Chang et al. (2019) say cognitive impairment and motor dysfunction can be induced by ROS‐induced neuronal cell death. Plant‐derived chemicals are gaining attention as possible neuroprotective drugs because of their antioxidant activity. Specifically, resveratrol and anthocyanins, two polyphenols highly present in berries and other fruits, have shown promise in promoting neurogenesis and reducing oxidative brain damage (Tauchen et al. 2016). By optimizing cell repair mechanisms and modifying inflammatory processes, Salehi et al. (2020) cited that daily consumption of antioxidant fruits may halt the course of neurodegenerative diseases (Salehi et al. 2020). On top of that, they have the ability to cure neurodegeneration induced by oxidative stress since they are capable of crossing the blood–brain barrier. The efficacy of antioxidants and regulation of oxidative stress levels are influenced largely by the gut microbiota, as reported by recent studies (Choque Delgado et al. 2023).

As per Agustinah et al. (2020), certain of these antioxidant short‐chain fatty acids are produced by the vigorous gut microbiota. These fatty acids have the competence of directly altering the body's level of oxidative stress. The current investigation exposed that joint use of healthy gut microbiota and antioxidant food consumption has been described to boost general well‐being and can help decrease the onset of diseases associated with oxidative stress (Naliyadhara et al. 2023). The obligation of using a holistic approach when dealing with oxidative stress is emphasized through the engagement among antioxidants and gut bacteria. Persons can support their microbiota in upholding a strong balance of pro‐ and anti‐inflammatory signals and avert oxidative impairment directly by consuming foods rich in antioxidants. Rendering to Choque Delgado et al. (2023), a pragmatic approach to regulate and avert diseases related to oxidative stress may be eating fruits and vegetables with greater antioxidant contented. Antioxidants decrease the interconnection of illnesses such as cancer, neurological sicknesses, and cardiovascular‐associated diseases by decreasing oxidative impairment (Avila‐Sosa et al. 2019). There is much indication that these bioactive particles can be therapeutic and that it is valuable to integrate them into one's diet for general long‐term health (Riaz Rajoka et al. 2021). Supplementary examination of these novel antioxidants and how they interconnect with other biological methods will expand our knowledge of their beneficial approaching and health belongings.

### 3.2 Anti‐Inflammatory Effect

**FIGURE 2:**
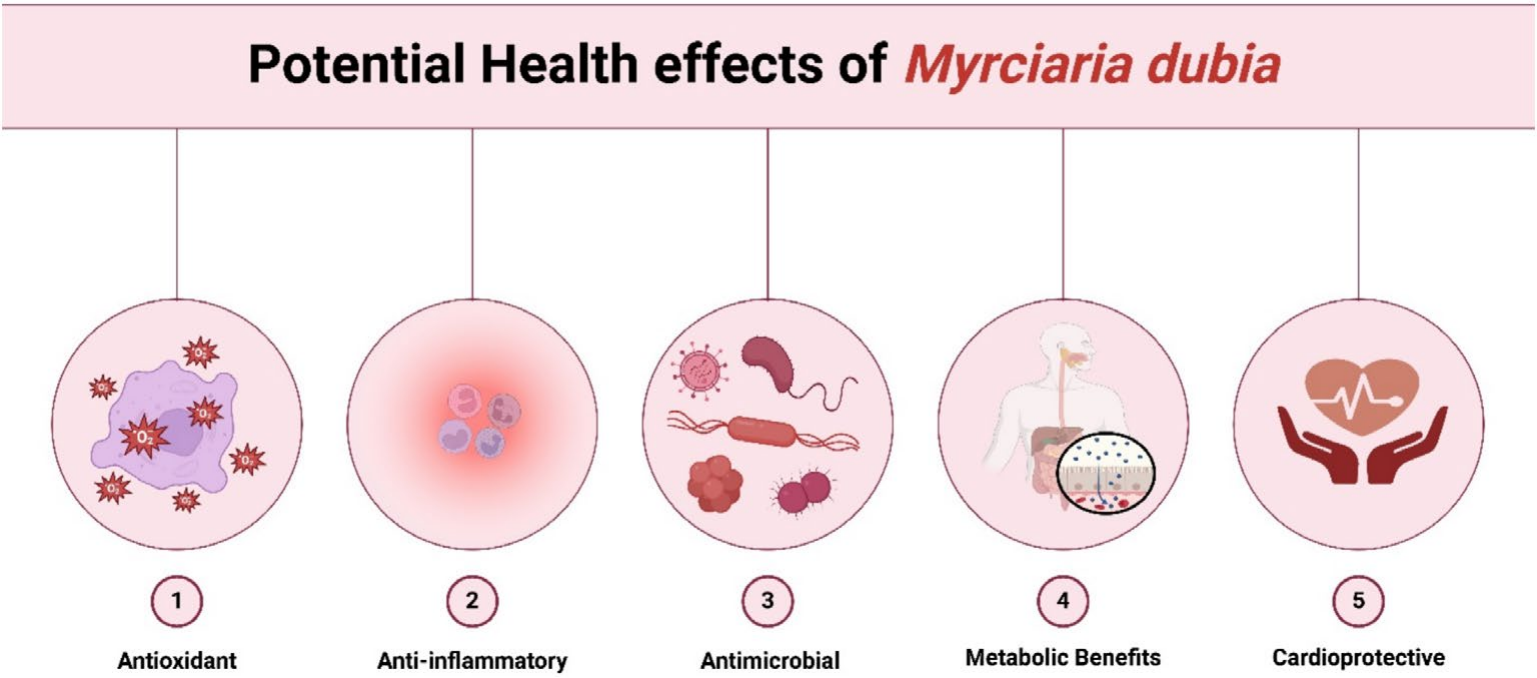
Potential health effects of *Myrciaria dubia*.

Found naturally in the Amazon, *Myrciaria dubia* is a slightly sour fruit. Greater nutritional and therapeutic potentials, according to Lima et al. (2020), are what describe Lima and its reputation. *Myrciaria dubia*'s anti‐inflammatory possessions make it a beneficial natural treatment for a diversity of inflammatory illnesses (Fidelis, de Oliveira, et al. 2020; Fidelis, do Carmo, et al. 2020), one of which is among its most significant health aids (Figure 2). It notes *Myrciaria dubia*'s anti‐inflammatory properties as well as its bioactive compounds, modes of action, and pharmacological marks for treating inflammation in syndromes (Lima et al. 2024). *Myrciaria dubia*'s active compounds, which are vitamin C, flavonoids, and polyphenols, are found in greater concentrations. These compounds persuade one of anti‐inflammatory action. Ironically, in *Myrciaria dubia*, vitamin C is a sturdier antioxidant that is operative at neutralizing oxidative stress, a key reason for inflammation (Lima et al. 2020). This antioxidant vitamin C (Fidelis, de Oliveira, et al. 2020; Fidelis, do Carmo, et al. 2020) balances free radicals, which are reactive particles that could trigger inflammatory pathways and impair tissues.

Also, the anthocyanins and other polyphenols and flavonoids of *Myrciaria dubia* can moderate inflammatory mediators, comprising prostaglandins and cytokines, which are main to the inflammatory process (García‐Chacón et al. 2023). Polyphenols, catechins, and ellagic acid are a limited number of the fruit's ingredients that decrease inflammatory markers in certain in vitro and animal models. These substances overwhelm inflammatory processes, particularly the MAPK and NF‐κB cascades, which subsequently abate inflammation (Castro et al. 2018; García‐Chacón et al. 2023). Lima et al. (2023) described that the bioactive compounds of *Myrciaria dubia* overwhelm proinflammatory cytokine production and other compounds that have effects on chronic inflammation by constraining these processes.

Most of the anti‐inflammatory activity of *Myrciaria dubia* consequences from its antioxidant prospective. To control oxidative stress, the vitamin C content of the fruit is essential. According to de Carvalho‐Silva et al. (2014), inflammation has a robust relation with oxidative stress. Different Koufan et al. (2024), the protracted diseases counting arthritis, cardiovascular disease, and neurological disorders, may be persuaded by long acquaintance with reactive oxygen species (ROS). Oxidative stress forms ROS, which also has a role in activating inflammation and impairment to tissues. *Myrciaria dubia* meaningfully decreases oxidative stress and inflammatory reactions by eradicating these reactive oxygen species (ROS.). Second, via altering the inflammation gene expression, the polyphenolic amalgams recognized in *Myrciaria dubia* show anti‐inflammatory action (Serrano et al. 2018). For instance, different examinations have revealed that by decreasing the COX‐2 enzyme activity, flavonoids comprised in this fruit are capable of overcoming the aggregation of inflammatory prostaglandins. Resting COX‐2 activity aids in decreasing pain and inflammation, conferring to Fidelis, de Oliveira, et al. (2020); Fidelis, do Carmo, et al. (2020).

Particularly for numerous inflammatory illnesses, *Myrciaria dubia*'s anti‐inflammatory possessions demonstrates tremendous therapeutic promise (de Carvalho‐Silva et al. 2014). Manica‐Cattani et al. (2022) exposed that *Myrciaria dubia* might be cooperative in treating autoimmune illnesses comprising RA. Pain, inflammation, and long‐term, combined degradation define chronic inflammatory arthritis (RA). *Myrciaria dubia*'s antioxidant and anti‐inflammatory properties should help RA patients restore their quality of life, based on García‐Chacón et al. (2023), by reducing disease activity and inflammatory response. Another condition demonstrating chronic inflammation of the gastrointestinal system is inflammatory bowel disease (IBD), which comprises two syndromes: ulcerative colitis and Crohn's disease. Mainly its flavonoids and polyphenols, the anti‐inflammatory bioactive compounds in *Myrciaria dubia* can assist in averting intestinal mucosal damage and regulating gut inflammation (Langley et al. 2015). By regulating inflammation and immunological reactivity through the preservation of homeostasis of gut bacteria, the compounds can be advantageous in upholding gut health according to Carmo et al. (2019).

*Myrciaria dubia* also demonstrates potential in the treatment of inflammatory illnesses of the cardiovascular system. The main reason for atherosclerosis and other cardiovascular disorders (Do et al. 2021) is long‐lasting low‐grade inflammation. In anticipation of cardiovascular events like heart attacks and strokes, the bioactive compounds identified in *Myrciaria dubia*, as reported by Abot et al. (2022) have the capability to lessen oxidative stress and endorse proinflammatory cytokines. Moreover, its capability to subordinate blood pressure and improve lipid profiles makes it an attractive tool for the management of cardiovascular diseases (Fidelis, de Oliveira, et al. 2020; Fidelis, do Carmo, et al. 2020). Although *Myrciaria dubia* has been revealed to have its own anti‐inflammatory properties in numerous investigations, supplementary clinical trials are desirable to approve its efficiency in humans. Even with promising results from first in vitro and animal studies, human trials are required to authenticate these results and ascertain ideal dosages (De Souza Schmidt Goncalves et al. 2010). Future (García‐Chacón et al. 2024) examination of the long‐term effects of supplementing with *Myrciaria dubia* ingestion, chronic inflammatory disorders, and the mechanisms involved in these diseases must be accompanied. In addition, there might be interest in the possible synergistic effects of *Myrciaria dubia* in association with other natural anti‐inflammatory drugs. The therapeutic benefits of *Myrciaria dubia* could be enhanced by combining it with other food items or dietary supplements rich in bioactive compounds. They, therefore, may offer a more potent natural anti‐inflammatory treatment (Agbaje et al. 2022). The complex profile of phytochemicals in *Myrciaria dubia*, vitamin C, flavonoids, and polyphenols is a strong natural reservoir for anti‐inflammatory compounds; hence, together, these act as modifiers of inflammatory pathways and diminish oxidative stress, which contributes significantly to the pathogenesis of most inflammatory diseases (de Carvalho and Conte‐Junior 2021). Although more clinical studies may still be required, *Myrciaria dubia* shows considerable potential for its treatment as a natural anti‐inflammatory agent, considering existing data. Incorporating *Myrciaria dubia* can provide promising supplementary therapies for rheumatoid arthritis, inflammatory bowel diseases, and cardiovascular conditions (Table 3) (Lima Tribst et al. 2009).

**TABLE 3:** Health benefits and therapeutic applications of *Myrciaria dubia*.

Effect	Dosage	Benefits	Reference
Antioxidant activity	2 g of Camu Camu powder daily	Enhanced antioxidant capacity in humans, helping reduce oxidative stress markers	Langley et al. (2015)
	100 mg/kg body weight (animal study)	Significant reduction in oxidative stress, lowering serum malondialdehyde levels	Fujita et al. (2015)
	50 mg/kg daily	Improved antioxidant defenses and reduced cellular oxidative damage in rats	Castro et al. (2020)
Anti‐inflammatory	200 mg/kg body weight (animal study)	Decreased inflammatory markers like TNF‐α and IL‐6 in obese rats	Abot et al. (2022)
	1 g of Camu Camu powder daily	Significant reduction in C‐reactive protein and other inflammatory markers	Fidelis, de Oliveira, et al. (2020); Fidelis, do Carmo, et al. (2020)
	2 g daily	Reduction of inflammation in chronic inflammatory diseases due to high vitamin C content	Langley et al. (2015)
Antimicrobial potential	50 mg/mL Camu Camu extract	Demonstrated antimicrobial activity against bacterial, yeast, and parasitic infections	Renteria et al. (2022)
	100 μg/mL extract	Inhibited growth of *Streptococcus mutans* , showing potential in oral hygiene applications	Camere‐Colarossi et al. (2016)
	500 mg/kg body weight	Reduction in microbial growth and improved infection control in infected rats	García‐Chacón et al. (2023)
Metabolic benefits	2 g daily	Significant reduction in body weight and improved lipid profile in obese rats	Carmo et al. (2019)
	100 mg/kg body weight (animal study)	Improved glucose metabolism and reduced insulin resistance in diabetic rats	Agrinier et al. (2024)
	200 mg/kg body weight (animal study)	Reduced obesity and improved cardiovascular profiles in obese diabetic mice	Abot et al. (2022)

### 3.3 Antimicrobial Potential

The Amazonian fruit *Myrciaria dubia*, which is great in vitamin C, is progressively anticipated due to its prospective solicitations in infection anticipation and food preservation (Castro et al. 2018). Numerous bioactive composites such as flavonoids, carotenoids, and phenolic acids are answerable for this fruit's antibacterial nature; it has formerly been mentioned to as *Myrciaria dubia* (Camere‐Colarossi et al. 2016). New investigation highlights these physiognomies, subsequently they communicate to the function of the fruit in circumventing food contamination by microbes and endorsing overall health. Numerous diseases, parasite protozoa, bacteria, and yeasts have been revealed to be reserved by *Myrciaria dubia*, based on investigation (Castro et al. 2020). For example, *Myrciaria dubia* established strong antibacterial activity in contradiction of *Salmonella* spp., *Staphylococcus aureus* , and *Escherichia coli* through a test of its prospective by Fujita et al. (2015). *Myrciaria dubia* is a main preserving because the stated bacteria are generally linked with food spoilage and food‐borne illness (Kaneshima et al. 2017). Even more significantly, Renteria et al. (2022) revealed that *Myrciaria dubia* extracts constrain food‐polluting yeasts like *Candida albicans* and *Saccharomyces cerevisiae* . The extraordinary polyphenolic contented of the fruit, which kills germs by breaking down their cell walls and membranes, is chiefly responsible for its antibacterial possessions. *Myrciaria dubia*'s antibacterial properties recognize it as a probable ingredient for food preservation approaches, which can extend the lifespan of perishable foodstuffs and render them harmless for consumption. Because further people need natural food additives, it may be a substitute for artificial preservatives (Gomes‐Rochette et al. 2016). There is a harmless and additional ecologically friendly replacement for toxic antimicrobial actions which can be a threat to resistance and health hazards that can be present in the bioactive composites of *Myrciaria dubia*. For instance, García‐Chacón et al. (2023) described that one can limit microbial infection by integrating *Myrciaria dubia* extracts into fruit juices, sauces, and dairy foodstuffs. Therefore, the shelf life of the products would be improved and spoilage abridged. Another novel method that is currently being discovered is the amalgamation of *Myrciaria dubia* into food packing materials. These ingredients have bioactive composites that are proficient in constraining the growth of microorganisms. One main concern concerning microbial pollution is the limited shelf life of poultry and meat foodstuffs; however, the antibacterial action of *Myrciaria dubia* could assist in this regard (Lima et al. 2020). Based on investigations, this fruit's extracts may be rummage sale as a natural dip or coating to reduce the number of microbes found in these products and have them more ecologically friendly than those with biochemical preservatives. The antibacterial potentials of *Myrciaria dubia* are not only advantageous for food preservation but also for the usage in combating illnesses, particularly in medicine. The effectiveness of this treatment in the anticipation and treatment of diverse infections, particularly drug‐resistant ones, is of thoughtful concern to medical specialists. Fidelis, de Oliveira, et al. (2020) and Fidelis, do Carmo, et al. (2020) deliberate *Myrciaria dubia*'s antimicrobial prospective in contradiction of the two most communal oral disease‐causing bacteria, *Streptococcus mutans* and *Streptococcus sanguinis* . As antibiotic conflict is rising more and more perilous internationally, natural antimicrobial composites like *Myrciaria dubia* hold promise as substitutions to conventional medications in the combat in contradiction of diseases. *Myrciaria dubia* has been investigated as an antibacterial agent in contradiction of cutaneous contaminations as well as for its advantageous effects on oral health. *Staphylococcus aureus* is accountable for most soft tissue infections and skin infections, but Camere‐Colarossi et al. (2016) study points out that *Myrciaria dubia* is an antibacterial mediator against *Staphylococcus aureus* . This suggests that a topical preparation of *Myrciaria dubia* can be advantageous against or in the treatment of skin infections. Camere‐Colarossi et al. (2016) hypothesized that *Myrciaria dubia* possesses a multi‐action antimicrobial mechanism. *Myrciaria dubia*, according to Camere‐Colarossi et al. (2016), endorses cell wall and membrane breakdown in microbial cells because of exposure to polyphenolic composites, particularly tannins and flavonoids. Cell death comes from this breakdown ultimately. By confining their metabolic activities, these amalgams can also slow down the outbreak of pathogenic bacteria. Based on investigations by Fujita et al. (2015), *Myrciaria dubia*'s high vitamin C concentration elucidates its antibacterial action. By varying oxidative stress in microbial cells and interrelating with free radicals, vitamin C has the capability to boost the immune reaction, conferring Wurlitzer et al. (2019). Recognitions to its antioxidant and antibacterial possessions, *Myrciaria dubia* is a robust food preservation and illness anticipation moderator. Based on positive results for food preservation and illness anticipations, *Myrciaria dubia*'s antibacterial activity still inspires tremendous expectation (Camere‐Colarossi et al. 2016). More investigation will help us regulate its behavior and what it can do when joined with other natural preservatives. Commercial uses demand further investigation on appropriate attention, extraction methods, and incorporation into food systems. Apart from its antibacterial action, the fruit possesses anti‐inflammatory and antioxidant properties that can further upsurge its capability to avert chronic diseases such as diabetes and cardiovascular disease (Luo et al. 2024). A versatile plant with numerous usages in the food and pharmaceutical sectors is *Myrciaria dubia* (da Silva et al. 2023). *Myrciaria dubia* is an all‐natural, ecologically safe food preservative and boasts robust antibacterial properties. *Myrciaria dubia* has potential in infection control and food safety as a consequence of its extensive spectrum antibacterial action. Predominantly, this is the case for food‐borne microorganisms and resistant kinds (da Silva et al. 2021). *Myrciaria dubia* can possibly play a significant role in contemporary efforts to preserve food safety and improve public health, but this will be contingent on the consequence of further studies into its full potential.

### 3.4 Metabolic Benefits

*Myrciaria dubia* plant has newly expanded in popularity due to the medical assistance of bioactive composites from the plant (Balisteiro et al. 2017). The numerous medical solicitations of *Myrciaria dubia* have been reputable for a long time. Liu et al. (2021) recognized that the development of metabolic health is one of the extensively rummaged benefits, particularly for the treatment of cardiovascular illness, diabetes, and obesity. *Myrciaria dubia* owns anti‐inflammatory, antioxidant, and insulin‐sensitizing properties, which it employs to improve metabolic homeostasis (Kojadinovic et al. 2017). The solicitation of *Myrciaria dubia* in the management of obesity has newly been the emphasis of heightened investigation, with promising consequences (Régnier et al. 2020). Methodical evidence specifies that the high quantities of antioxidants in the fruit, particularly vitamin C and polyphenols, affect the pathways in the metabolism, leading to fat storing and fat cell function, or adipocytes. *Myrciaria dubia*, as per Silva (2022), can contribute to humans in weight loss by aggregating the activity of metabolic regulators. For example, animal investigations have revealed that the metabolic profiles and percentage of body fat of obese mice were improved when directed *Myrciaria dubia* extract supplements (Fidelis, de Oliveira, et al. 2020; Fidelis, do Carmo, et al. 2020; García‐Chacón et al. 2023).

The fruit is supposed to affect the metabolism, energy spending, and obesity of persons (Balisteiro et al. 2017). Additionally, attention to the influence of the gut microbiota on the treatment of obesity is cumulative, and *Myrciaria dubia* may be capable of making an influence on this (Noguera et al. 2024). The positive influences of food polyphenols on microbiota, metabolic processes, and weight control have been recognized very well in a number of investigations. Obesity is typically linked with modifications in the gut microbiota (García‐Chacón et al. 2023). The effects of *Myrciaria dubia* on glucose metabolism have been inspected in both rat and human investigations. Augmented insulin sensitivity and reduced blood glucose have been associated with the extraordinary vitamin C and other polyphenolic compounds found in the fruit. Consequences exposed that diabetic mice supplemented with *Myrciaria dubia* experienced meaningfully fewer hyperglycemia episodes and enhanced insulin resistance (Abot et al. 2022). These results designate that *Myrciaria dubia* can interact with insulin signaling pathways and subsequently could be an outstanding addition to Type 2 diabetes drugs. One of the main reasons for insulin resistance among diabetes patients is chronic low‐grade inflammation; this fruit can reduce inflammation due to its anti‐inflammatory properties (Langley et al. 2015). *Myrciaria dubia* supplementation has been revealed to progress blood glucose regulation, decrease biomarkers related to diabetic complications, and decrease oxidative stress in human trials (Barbalho et al. 2021). These properties are thought to be enhanced by the extraordinary levels of polyphenols, which upsurge the antioxidant defenses of the body and decrease oxidative stress. Oxidative stress feeds insulin resistance and the failure of pancreatic beta cells.

### 3.5 Cardioprotective Effect

The positive influences of *Myrciaria dubia* on cardiovascular health are mainly accredited to its ironic antioxidant content (da Silva et al. 2021). Anhê et al. (2019) stipulate that the fruit contains polyphenols and flavonoids, elements well known to deliver protection against oxidative stress and inflammation. The chief causes of cardiovascular disease are these ones. *Myrciaria dubia*, conferring to García‐Chacón et al. (2023), drops the level of oxidative damage triggered on endothelial cells thereby refining the function of blood vessels. Moreover, investigations have found that *Myrciaria dubia* drops blood pressure, which is a significant factor manipulating the upsurge in cardiovascular disease. The fruit could be valuable in controlling hypertension (Langley et al. 2015; Abot et al. 2022) because of its capability to fight inflammation and oxidative stress, which have been revealed to impair vascular function and upsurge blood pressure jointly. *Myrciaria dubia*'s great vitamin C concentration has been revealed in accumulation to subordinate LDL cholesterol levels, thus restraining atherosclerosis and general heart health (Castro et al. 2018). Two ways *Myrciaria dubia* can aid guard against cardiovascular disease, which consists of coronary heart disease that has abridged oxidized LDL levels and better cholesterol profiles. Metabolic diseases such as diabetes, obesity, and cardiovascular illness have a lot of prospective when they are treated with the functional mealtime *Myrciaria dubia*. Improved metabolic health is achieved through the anti‐inflammatory, antioxidant, and insulin‐sensitizing properties of the polyphenols and vitamin C that are found in bioactive composites (da Silva et al. 2012). There is growing indication that the fruit has the capability to regulate metabolism, demonstrating that it might be advantageous as a natural medicine for the anticipation and treatment of long‐lasting diseases, particularly those linked with metabolic derangement (Dutta et al. 2018).

## 4 Bioavailability and Efficacy of *Myrciaria dubia*

*Myrciaria dubia* appreciates a number of assistances with regard to bioavailability and effectiveness, joint with its high content of bioactive constituents (Camere‐Colarossi et al. 2016). A food supplement's “bioavailability” is its capability to be engrossed and exploited by the body. The antioxidant effect is accomplished by the body's operative absorption and exploitation of vitamin C, which is found in high levels in *Myrciaria dubia*, as emphasized by García‐Chacón et al. (2024). It contributes to cellular inflammation and oxidative stress mitigation due to its bioactivity and other bioactive composites, comprising polyphenols and flavonoids (Zhang et al. 2023; Santos et al. 2022). The polyphenolic composites in *Myrciaria dubia* are also fairly accessible, with a comparatively high percentage; this is because it is soluble in water, which increases its uptake in the digestive system (Fujita et al. 2015). These composites, when supplemented with vitamin C, deliver an integrated method toward enhanced health through an improved immune system, a decrease in inflammation, and oxidative stress. Almeida et al. (2024) described that *Myrciaria dubia* is extra bioavailable and powerful when consumed on a consistent basis due to the greater concentration of water‐soluble bioactive composites. In scientific trials, *Myrciaria dubia* has produced encouraging consequences, particularly in reducing oxidative stress and inflammation markers. These composites are accountable for prolonged diseases such as cancer and cardiovascular disease, and *Myrciaria dubia* has this advantage over other fruits such as açaí and blueberries (Fujita et al. 2015). *Myrciaria dubia* is among bioactive fruits that are distinguished for their vitamin C content, antioxidant power, and well‐adjusted flavonoid and polyphenol profiles.

Certain superfruits, such as acerola, açaí, and blueberries, are less dense in bioactive substances and have lesser bioavailabilities than *Myrciaria dubia*. Its antioxidant and anti‐inflammatory properties are in large part the consequence of the high concentrations of vitamin C, flavonoids, and polyphenols that it possesses. The high concentrations of water‐soluble bioactive compounds in the fruit assist absorption and utilization by the body, increasing the bioavailability and efficiency of the fruit (Kaneshima et al. 2017). *Myrciaria dubia* has a massive potential to add to the benefits of healthy diets, bearing in mind the attention in superfruits and functional foods round the globe, according to Patel et al. (2018).

## 5 Industrial Applications of *Myrciaria Dubia*

**FIGURE 3:**
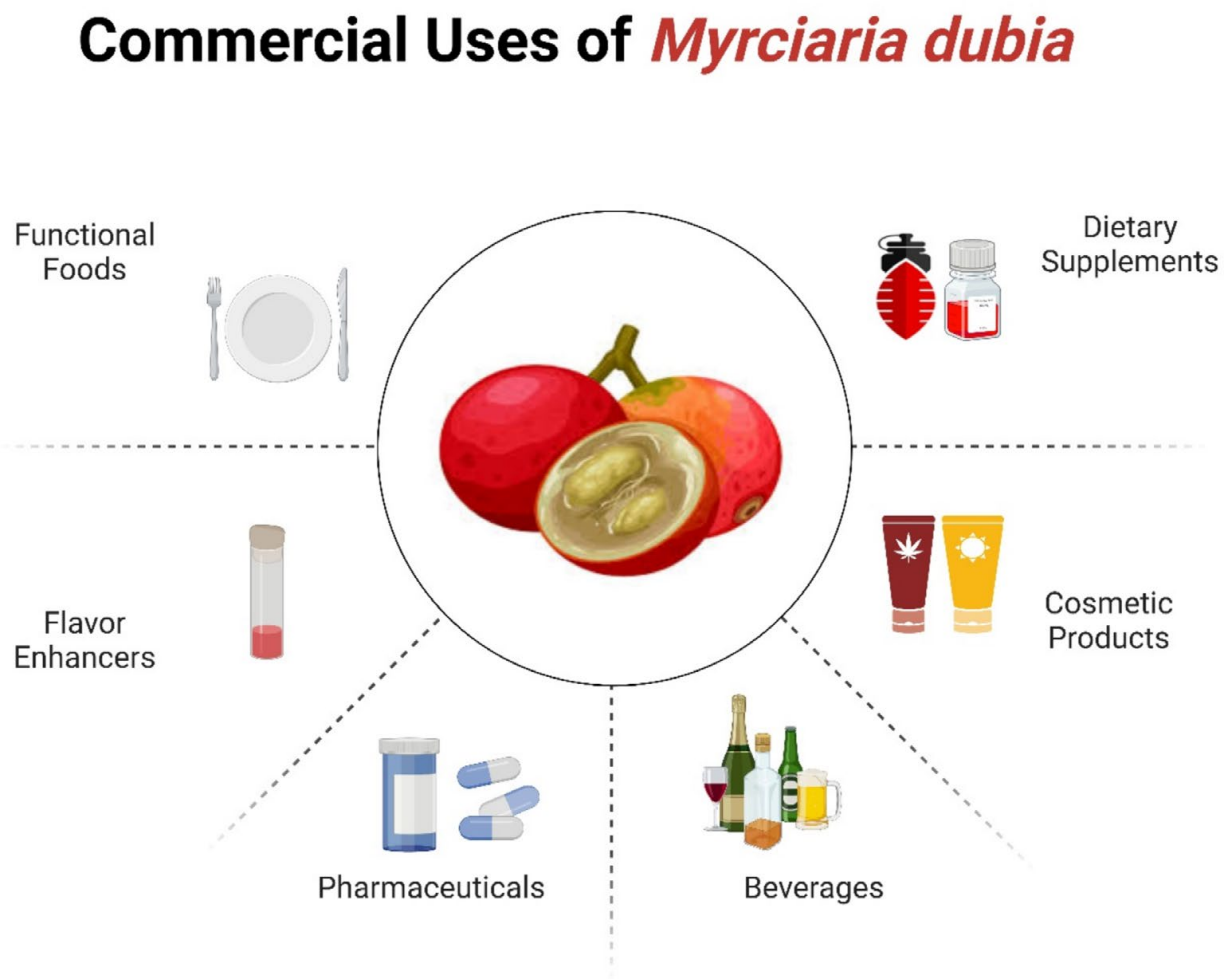
Commercial uses of *Myrciaria dubia*.

Rendering to Araujo et al. (2024), *Myrciaria dubia* comprises a lot of valuable composites such as vitamin C, anthocyanins, and flavonoids. As per Santos et al. (2022), it comprises strong antioxidant and anti‐inflammatory properties. As is evident from Figure 3, its usefulness in the functional food and beverage manufacturing is supplemented by its health‐giving properties, which are anticipated by customers. The increasing awareness of customers about the health benefits of specific foods is fueling a boom in petitions for the worldwide functional food marketplace (García‐Chacón et al. 2023). Pinela et al. (2016) exposed that the great content of bioactive composites in *Myrciaria dubia* makes it a good contender for a functional meal revolution. To improve skin tightness, immunological health, and general wellness, it is normally added to juices, smoothies, and dietary supplements due to its antioxidant properties (Castro et al. 2018). Organic or natural component beverages like *Myrciaria dubia* are progressively sought after as an additive or boosting mediator (Oliveira et al. 2024). Energy bars, snack foods, and meal replacement foods are merely a few solicitations of the diversity of functional food goods that are probable through mixtures of *Myrciaria dubia* with other superfoods. This, rendering to Azevedo et al. (2019), generates new avenues for development in the trade. de Abreu Figueiredo et al. (2020) stated that its ironic vitamin C content and anti‐inflammatory and antioxidant properties make it a perfect component for products that improve general strength, skin well‐being, and immune resilience.

## 6 Pharmacological Effects of *Myrciaria dubia*

**FIGURE 4:**
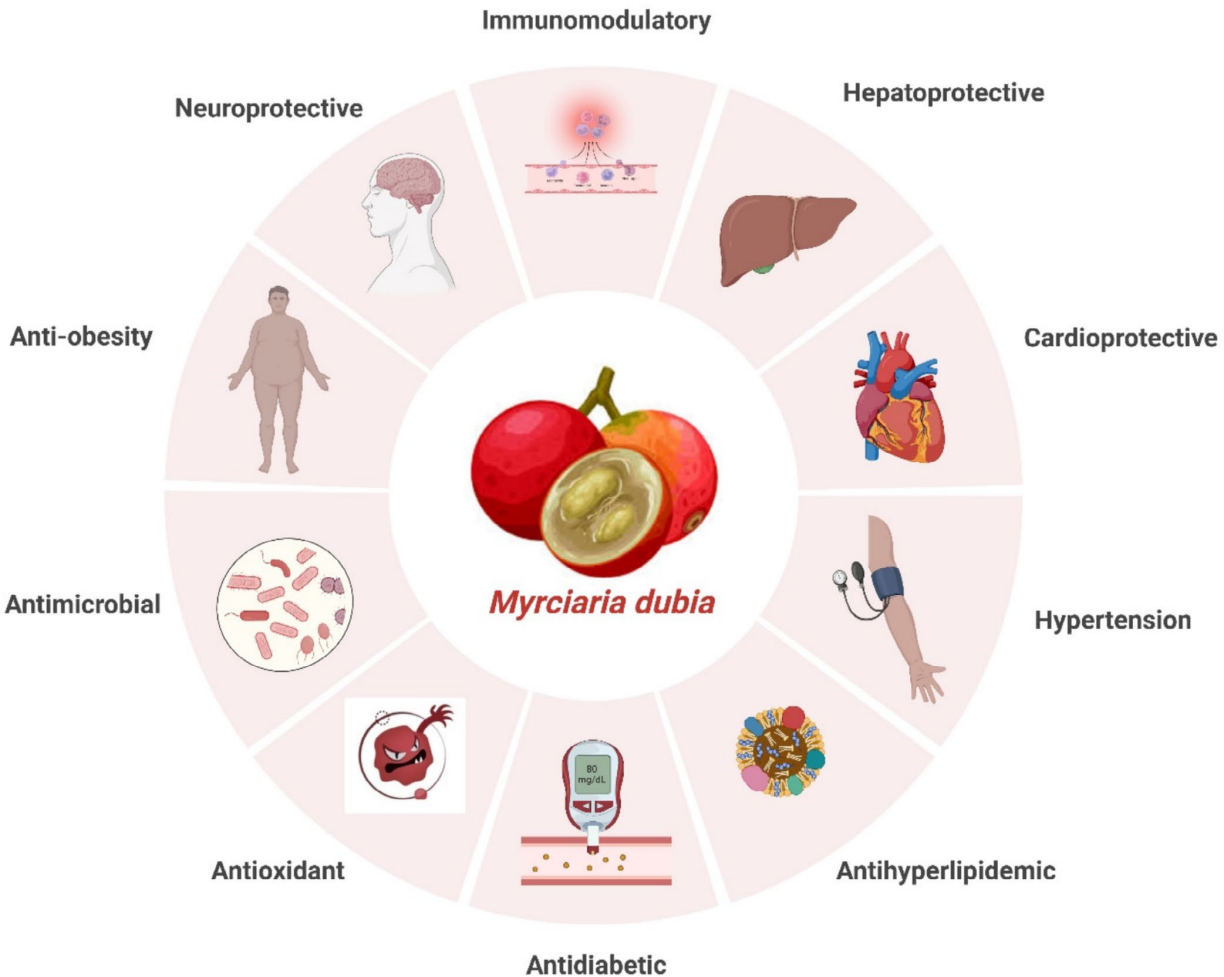
Therapeutic effects of *Myrciaria dubia*.

*Myrciaria dubia* is very auspicious in the nutraceutical and pharmaceutical business, as well as in functional foods and makeup (Anaya‐Esparza et al. 2020). *Myrciaria dubia*'s medicinal prospective is due to its bioactive composites, which are vitamins, phenolic acids, and flavonoids; certain of these composites have been investigated in numerous parts of medicine (de Abreu Figueiredo et al. 2020). Among the long‐standing disorders *Myrciaria dubia* is adept at regulating are hyperglycemia, diabetes, and hypertension (Figure 4). Rendering to experts, the bioactive composites present in this fruit have prospective to lesser blood sugar levels, subordinate inflammation, and lesser hypertension (Inoue et al. 2008). This permits nutraceutical strategies to aid both metabolic diseases and cardiovascular health to be challenged. Given its antibacterial approaching (Fidelis, de Oliveira, et al. 2020; Fidelis, do Carmo, et al. 2020), treatments for infections or immune system regulation could be sensibly feasible. Exploration of the anticancer possessions of *Myrciaria dubia* extract is still in progress. While oxidative stress is the main cause of numerous cancers' progressive character, this fruit's great antioxidant properties help to eliminate them (Santos et al. 2022). *Myrciaria dubia* may be used as an adjuvant to cure cancer due to its components, which are thought to be antimutagenic and inhibitory to the development of malignant cells (Azevedo et al. 2019). Another sector of possible medical usage for *Myrciaria dubia* is using it to promote cellular regeneration. Investigation by Fujita et al. (2015) demonstrates that *Myrciaria dubia* can restore cellular activity and protect against oxidative damage, which is connected with aging and degenerative illnesses. With its broad‐ranging bioactive profile, *Myrciaria dubia* can be a beneficial and safe component in pharmaceutical products for the prevention or treatment of prevalent multiple diseases (Table 4).

**TABLE 4:** Functional and nutritional properties of *Myrciaria dubia* and key challenges.

Study	Application	Dosage	Effect	Key challenges
Fujita et al. (2015)	Functional foods and beverages	500 mg/kg	Antihyperglycemic and antihypertensive effects in animal models	Lack of human clinical trials
Azevedo et al. (2019)	Functional foods and beverages	50–100 mg/mL of Camu Camu extract	Higher antioxidant capacity and bioactive compound content	Variability in bioactive compound concentration due to cultivation conditions
García‐Chacón et al. (2024)	Functional foods and beverages	1–2 g of Camu Camu powder	Bioactive compound profile; antioxidant and antimicrobial properties	Processing methods affecting nutrient stability
Marina et al. (2024)	Cosmeceuticals	10%–20% Camu Camu extract	Enhanced epidermal barrier function, antiaging effects in ex vivo human skin models	Limited commercial formulations available
Inoue et al. (2008)	Cosmeceuticals	100 mg/kg	Anti‐inflammatory, antioxidant properties; potential for skincare	Stability of bioactive compounds in topical applications
Fidelis, de Oliveira, et al. (2020); Fidelis, do Carmo, et al. (2020)	Pharmaceuticals	500 mg/kg	Antioxidant, antihyperglycemic, antiproliferative, antimicrobial, and anti‐inflammatory properties	Bioavailability and formulation challenges
Santos et al. (2022)	Pharmaceuticals	10–50 mg/mL of extract	In vitro cytotoxicity against cancer cells; antibacterial properties	Lack of standardized extract concentrations
Fujita et al. (2017)	Pharmaceuticals	200–300 mg of Camu Camu extract	Cellular rejuvenation, antioxidative, and anti‐inflammatory effects	High cost of extraction and processing
Castro et al. (2018)	Functional foods and beverages	1–2 g of Camu Camu powder	High levels of antioxidants and polyphenols	Limited shelf life of fresh fruit
Kino et al. (2020)	Functional foods and beverages	100 mg/day of extract	Improved gut health and modulation of gut microbiota	Need for more human clinical evidence
Stefanello et al. (2023)	Cosmeceuticals	10% Camu Camu cream	Improvement in skin elasticity and reduced wrinkle formation	Need for stability studies in cosmetics
Pacheco et al. (2020)	Pharmaceuticals	100 mg/mL of extract	Anti‐inflammatory effects in chronic conditions	Standardization of dosing in pharmaceuticals
López et al. (2016)	Functional foods and beverages	500 mg/kg of Camu Camu extract	Enhances antioxidant defense systems, increases vitamin C levels	Seasonal variability in fruit composition
Mishra et al. (2022)	Cosmeceuticals	15% Camu Camu extract	Skin hydration, antiaging, and enhanced collagen synthesis	Need for deeper dermatological research
Batista et al. (2021)	Functional foods and beverages	300 mg of Camu Camu extract	Positive impact on liver health and metabolism	Regulatory approval for functional claims
Ribeiro et al. (2018)	Pharmaceuticals	500 mg/day	Anticancer properties against lung and colon cancer cell lines	Lack of large‐scale human trials
Rodrigues et al. (2020)	Cosmeceuticals	20% Camu Camu serum	Skin repair and regeneration; enhanced epidermal health	Limited penetration in global skincare markets
Santos et al. (2022)	Pharmaceuticals	100–300 mg/day	Improvement of cognitive function and anti‐inflammatory effects	Need for long‐term safety studies

*Myrciaria dubia* is exploited as a constituent of plentiful pharmaceuticals, cosmeceuticals, and functional yields due to the greater content of bioactive composites. Its antioxidant, anti‐inflammatory, and antibacterial actions serve as a solid basis for the advancement of health‐supportive foodstuffs. The food industry previously exploited *Myrciaria dubia* to produce beverages and dietary supplements that upkeep overall health and immunity. A vital part of cosmeceuticals in the cosmeceutical section of the cosmetics industry, it improves skin health and postpones aging. Its potential as a pharmaceutical and nutraceutical beneficial product reflects the future of improving health and managing prolonged diseases. Aggregate numbers of persons seek natural, helpful products, so the marketplace for *Myrciaria dubia* foodstuffs will grow.

Pharmaceutical and cosmetically vigorous cosmetics found a speedily increasing industry under the cosmeceutical skincare group (de Abreu Figueiredo et al. 2020). Due to its high concentration of influential antioxidants, including vitamin C and phenolic composites, *Myrciaria dubia* has outstanding potential as an active component in antiaging and skincare goods (Langley et al. 2015). *Myrciaria dubia* has potential as a skincare item because it can counteract free radicals, which cause skin impairment and aging (Fujita et al. 2015). Collagen is a protein that upholds the firmness and wrinkle‐free form of skin, and a profusion of vitamin C in the diet can certify its production. Because of its anti‐inflammatory and antibacterial properties, it supports the treatment of skin conditions like psoriasis, eczema, and acne (García‐Chacón et al. 2024). Additionally, this will keep the skin in opposition to environmental aggressors such as pollution and UV radiation, so the antioxidant content in the fruit is vital. More and more antiaging, improving, and moisturizing modern creams, serums, and masks comprise *Myrciaria dubia*. For instance, Marina et al. (2024) reported recently that a dermatological product that comprises ceramide, inspiring peptides, and *Myrciaria dubia* extract reinforces the antiaging properties of the skin and the epidermal barrier function. These developments demonstrate that the fruit can be engaged in high‐end cosmeceuticals with emphasis on skin renewal and antiaging.

## 7 Future Research Directions and Limitations

Though the prevailing investigation highlights the prospective of *Myrciaria dubia* (camu‐camu) as a functional food, numerous areas endure underexplored. Future investigation should pay attention to heightening extraction approaches to make the most of the retention of bioactive composites, such as polyphenols and vitamin C, through processing (Cunha‐Santos et al. 2022; da Silva et al. 2023). Moreover, the mechanisms underlying *Myrciaria dubia* antioxidant, anti‐inflammatory, and antidiabetic properties permit additional examination, predominantly in humanoid clinical trials, to authenticate preclinical outcomes (Fidelis, de Oliveira, et al. 2020; Fidelis, do Carmo, et al. 2020; Do et al. 2021). The synergistic properties of *Myrciaria dubia* with further functional foods or nutraceuticals could also be discovered to improve its health assistance (Conceição et al. 2019; de Abreu Figueiredo et al. 2020). Also, an investigation should address the bioavailability of *Myrciaria dubia* bioactive composites and their prolonged special effects on chronic illnesses, such as obesity, diabetes, and cardiovascular disorders (Donado‐Pestana et al. 2018; Nascimento et al. 2013). Finally, the ecological and economic sustainability of *Myrciaria dubia* farming and processing should be assessed to support its profitable feasibility and accessibility (García‐Chacón et al. 2023; Oliveira et al. 2024). Addressing these breaches will advance the applicability of *Myrciaria dubia* as a functional foodstuff and increase its prospective in worldwide health and nutrition (Zhang et al. 2021).

## 8 Conclusion and Future Perspectives

The exceptional bioactive conformation of *Myrciaria dubia*, such as a varied range of polyphenolic composites and a record vitamin C content, renders it a strong functional food composite with frequent health assistance. Its metabolic, anti‐inflammatory, and antioxidant activities point to its prospective in the anticipation and treatment of prolonged diseases. *Myrciaria dubia* has confirmed itself to be a valued, maintainable agricultural product and natural remedy by surpassing other fruits in bioavailability and beneficial activity, as demonstrated by comparative analysis. However, there is much unrealized prospective owing to its current underutilization and deficiency of adequate incorporation into mainstream health yields. To preserve the bioactive physiognomies of the fruit and harvest its prospective, there is a requirement for new maintainable production and processing techniques. Filling investigation gaps, chiefly in clinical trials, is significant to endorse its therapeutic usages and control its feasibility for the pharmaceutical, cosmeceutical, and nutraceutical businesses.

The fabrication and processing of *Myrciaria dubia* can be reformed by biotechnological progressions, which will raise its bioactive value and certify that the same level of quality is preserved continually. Agroforestry system regulations, genetic reform, and Amazonian biodiversity conservation can improve yields. The added benefit is that the bioactive and nutritional content of the fruit is preserved by novel processing methods such as low‐temperature drying and green extraction technologies, which makes it more sellable internationally. *Myrciaria dubia* has a bright future in nutraceutical and functional foods; however, its well‐being will depend on careful marketing, awareness‐raising efforts, and legislative support. Together with researchers, legislators, and local communities, the fruit's contribution to global health problems and fair advantages for native populations can be ensured by a guaranteed system of sustainable farming methods. This super fruit is also a symbol of biodiversity preservation and sustainable development.

## Author Contributions

**Sammra Maqsood:** methodology (equal), writing – original draft (equal). **Muhammad Tayyab Arshad:** data curation (equal), writing – review and editing (equal). **Ali Ikram:** supervision (equal), validation (equal). **Kodjo Théodore Gnedeka:** conceptualization (equal), project administration (equal).

## Disclosure

The authors have nothing to report.

## Ethics Statement

This study did not involve humans or animals.

## Consent

This study did not involve humans.

## Conflicts of Interest

The authors declare no conflicts of interest.

## Data Availability

The data supporting this study's findings are available from the corresponding author upon reasonable request.
